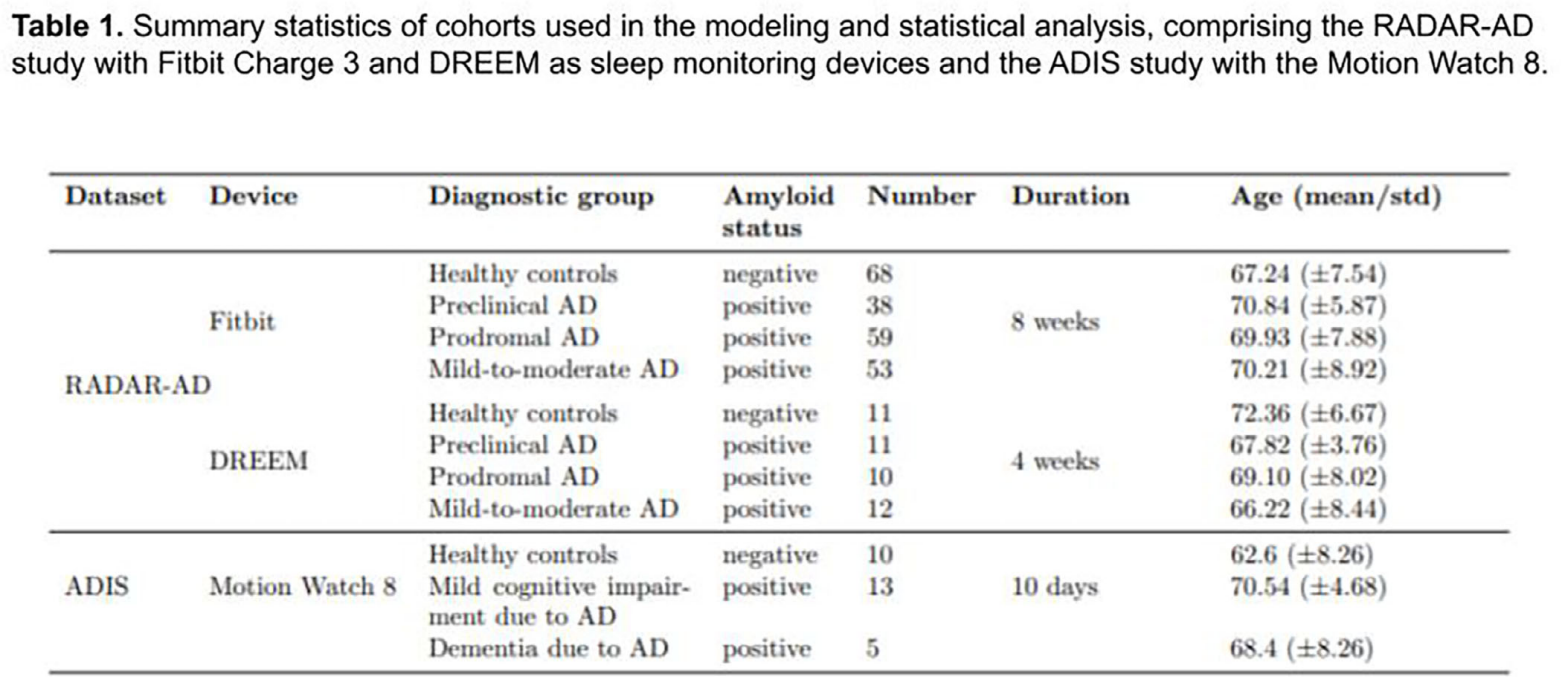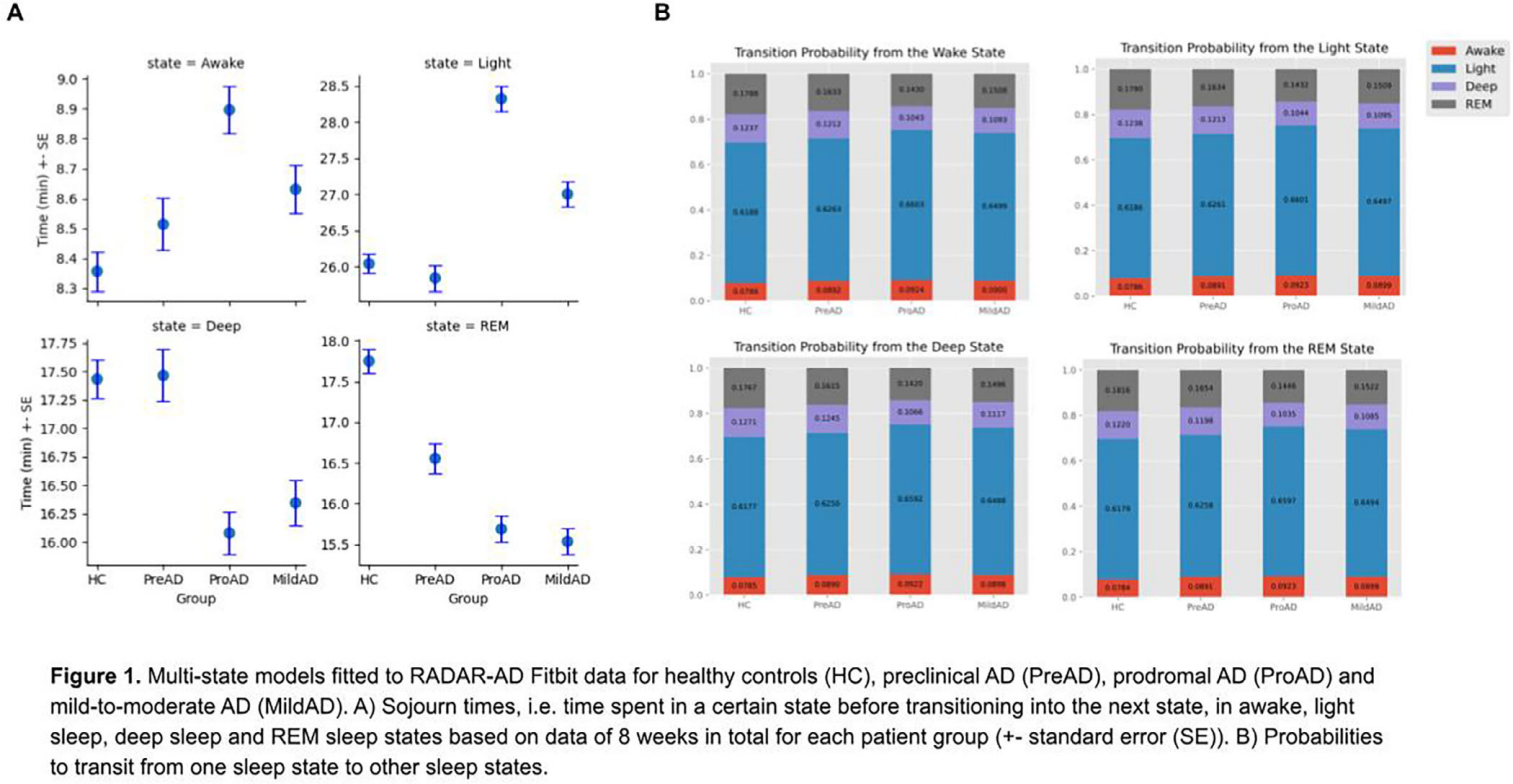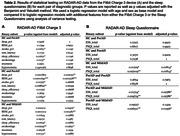# Digitally Assessed Sleep Disturbances for Early Diagnosis of Alzheimer’s Disease

**DOI:** 10.1002/alz.088269

**Published:** 2025-01-09

**Authors:** Sophia Krix, Joud Odeh, Manuel Lentzen, Andrea Val‐Guardiola, Neus Falgàs Martínez, Alankar Atreya, Pauline Conde, Aiden Doherty, Srinivasan Vairavan, Marijn Muurling, Casper de Boer, Jelena Curcic, Margarita Grammatikopoulou, Spiros Nikolopoulos, Anna‐Katharine Brem, Neva Coello, Vaibhav Narayan, Gayle Wittenberg, Cornelia M Van Duijn, Chris Hinds, Dag Aarsland, Raquel Sanchez‐Valle, Holger Fröhlich

**Affiliations:** ^1^ University of Bonn, Bonn, NRW Germany; ^2^ Fraunhofer Institute for Algorithms and Scientific Computing, Sankt Augustin, NRW Germany; ^3^ Fraunhofer SCAI, Sankt Augustin, NRW Germany; ^4^ Bonn‐Aachen International Center for IT (b‐it), Bonn Germany; ^5^ Fraunhofer Institute for Algorithms and Scientific Computing SCAI, Sankt Augustin Germany; ^6^ Alzheimer’s disease and other cognitive disorders Unit. Hospital Clínic de Barcelona. Fundació de Recerca Clínic Barcelona – IDIBAPS. University of Barcelona, Barcelona Spain; ^7^ Alzheimer's disease and other cognitive disorders unit, Hospital Clínic, IDIBAPS, Barcelona Spain; ^8^ University of Oxford, Oxford United Kingdom; ^9^ King's College London, London United Kingdom; ^10^ Janssen Research and Development LLC, Titusville, NJ USA; ^11^ Alzheimer Center Amsterdam, Neurology, Vrije Universiteit Amsterdam, Amsterdam UMC location VUmc, Amsterdam Netherlands; ^12^ Amsterdam Neuroscience, Neurodegeneration, Amsterdam Netherlands; ^13^ VU University Medical Center, Amsterdam Netherlands; ^14^ Novartis Institutes for BioMedical Research, Basel Switzerland; ^15^ Centre for Research & Technology Hellas, Thessaloniki Greece; ^16^ King’s College London, London United Kingdom; ^17^ Davos Alzheimers Collaborative, Wayne, PA USA; ^18^ Johnson & Johnson Innovative Medicine, Titusville, NJ USA; ^19^ Nuffield Department of Population Health, University of Oxford, Oxford United Kingdom; ^20^ King's College London, London, England United Kingdom; ^21^ Stavanger University Hospital, Stavanger Norway; ^22^ Alzheimer's Disease and Other Cognitive Disorders Unit, Hospital Clínic, Institut d'Investigacions Biomèdiques August Pi i Sunyer (IDIBAPS), Barcelona Spain; ^23^ Institut de Neurociències, Universitat de Barcelona, Barcelona Spain

## Abstract

**Background:**

Alzheimer’s Disease (AD) is associated with sleep disturbances. Moreover, individuals with sleep disturbances have been reported to have a higher risk for developing AD. The measurement of sleep behavior therefore opens the opportunity for a potential digital biomarker of AD.

**Method:**

We modeled sleep patterns coming from the RADAR‐AD cohort from two sleep monitoring devices (Table 1). We applied a stochastic modeling approach, multi‐state models, and analyzed the times spent in each sleep state before transitioning and transition probabilities in and between sleep states. We further applied statistical analysis of sleep monitoring data and sleep questionnaires (ESS, PSQI) from the RADAR‐AD study (Fitbit Charge 3, DREEM) and preliminary data from the ADIS study (MotionWatch8) (Table 1), using a likelihood ratio test with the aim to assess the diagnostic potential of sleep monitoring devices compared to traditional sleep questionnaires.

**Result:**

Modeling of digital device data showed that preclinial (preAD), prodromal (proAD) and mild‐to‐moderate (mildAD) AD patients spent more time in the light sleep and awake state, and less time in the REM sleep states compared to healthy controls (HC) before transitioning to the next state, showing non‐linear associations between diagnostic stage and sojourn times (Figure 1A). ProAD and mildAD patients had a higher probability to transit to a light sleep phase compared to HC and to subsequently wake up (Figure 1B). Based on our current and partially still preliminary data, only digital sleep monitoring via Fitbit allowed for a separation between HC and preclinical AD at nominal significance (preAD) but findings were not significant when adjusting for multiple testing (Table 2A). A significant distinction between HC and proAD (p < 0.001) as well as between HC and mildAD (p < 0.01) was possible using Fitbit sleep monitoring data, whereas traditional sleep questionnaires were only able to distinguish HC from mildAD (p < 0.05) (Table 2).

**Conclusion:**

Sleep patterns assessed via tested digital devices were able to separate proAD from HC – in case of Fitbit ‐ which was not possible by traditional sleep questionnaires. Digital sleep monitoring has thus the potential to support the early diagnosis of dementia.